# Specific Insect-Virus Interactions Are Responsible for Variation in Competency of Different *Thrips tabaci* Isolines to Transmit Different *Tomato Spotted Wilt Virus* Isolates

**DOI:** 10.1371/journal.pone.0054567

**Published:** 2013-01-24

**Authors:** Alana L. Jacobson, George G. Kennedy

**Affiliations:** Department of Entomology, North Carolina State University, Raleigh, North Carolina, United States of America; University of Oxford, United Kingdom

## Abstract

Local adaptation between sympatric host and parasite populations driven by vector genetics appears to be a factor that influences dynamics of disease epidemics and evolution of insect-vectored viruses. Although *T. tabaci* is the primary vector of *Tomato spotted wilt virus* (TSWV) in some areas of the world, it is not an important vector of this economically important plant virus in many areas where it occurs. Previous studies suggest that genetic variation of thrips populations, virus isolates, or both are important factors underlying the localized importance of this species as a vector of TSWV. This study was undertaken to quantify variation in transmissibility of TSWV isolates by *T. tabaci*, in the ability of *T. tabaci* to transmit isolates of TSWV, and to examine the possibility that genetic interactions and local adaptation contribute to the localized nature of this species as a vector of TSWV. Isofemale lines of *Thrips tabaci* from multiple locations were tested for their ability to transmit multiple TSWV isolates collected at the same and different locations as the thrips. Results revealed that the probability of an isofemale line transmitting TSWV varied among virus isolates, and the probability of an isolate being transmitted varied among isofemale lines. These results indicate that the interaction of *T. tabaci* and TSWV isolate genetic determinants underlie successful transmission of TSWV by *T. tabaci*. Further analysis revealed sympatric vector-virus pairing resulted in higher transmission than allopatric pairing, which suggests that local adaptation is occurring between *T. tabaci* and TSWV isolates.

## Introduction

The importance of compatible host and parasite genetics that result in infection are well documented for plant and mammalian pathosystems ([1], [2] and references therein). In these systems gene-for-gene or matching-allele interactions govern infectious interactions between hosts and parasites. Additionally, local adaptation between sympatric host and parasite populations driven by vector genetics appears to be a factor driving dynamics of disease epidemics and evolution of insect-vectored viruses, such as dengue viruses transmitted by mosquitoes [3], [4] and whitefly transmitted begomoviruses [5].

Thrips (Thysanoptera: Thripidae)-vectored viruses in the genus *Tospovirus*, the only plant-infecting members of the family Bunyaviridae, are rapidly emerging threats to a diversity of crops worldwide [6]–[8]. Eight *Tospovirus* species have been confirmed and an additional 16 emerging genotypes have been identified [8]. *Tomato spotted wilt virus* (TSWV), the type member of the genus, is a persistently propagative, thrips-transmitted, plant virus reported to infect over 900 different plant species including many economically important agricultural crops. During the 1980s significant spread of TSWV occurred globally due to the transport of infected material and vector species [7], [9], [10]. TSWV has been present in the southeastern USA, including North Carolina, since the 1980s, and frequently causes significant losses in tobacco, peanut, pepper, tomato, and potato [7], [11]. At least 10 thrips species are reported vectors of TSWV, but only three of these vectors are present in NC: *Thrips tabaci* Lindeman, *Frankliniella fusca* Hinds and *F. occidentalis* Pergande. Research on TSWV in the southeastern US has focused on *F. fusca* and *F. occidentalis* as vectors and has yielded important information about the epidemiology of the virus in NC [12]–[15], but information on *T. tabaci* is lacking.

*T. tabaci* has not been regarded as an important vector of TSWV in NC due to the localized and inconsistent presence of *T. tabaci* populations in NC agroecosystems, and the variability that exists in transmission efficiency in this species [16]–[25]. However, numerous years of thrips trapping data show that in some years, and in some locations where TSWV occurs, *T. tabaci* are caught in numbers similar to those of other vector species [26]. In addition, although variation in transmission efficiency has been documented in New York and North Carolina populations of *T. tabaci* [22], variable transmission efficiencies among populations are also observed in locations where *T. tabaci* is implicated as the primary vector species responsible for significant crop losses due to TSWV [19], [20], [23]–[25], [27].

Variation in transmission efficiency among *T. tabaci* populations has been associated with differences in reproductive mode (either arrhenotokous or thelytokous parthenogenesis) and host plant preferences [19], [28]. Viral factors responsible for variation in transmission have received less attention but it has been shown that a population of *T. tabaci* will transmit two different TSWV isolates with different efficiencies [29], [30]. Genetic factors underlying transmission are not well characterized, but a genetic basis for transmission has been identified in both TSWV and *T. tabaci*. Genetic backcross experiments between poor and efficiently transmitting populations of *T. tabaci* demonstrated that the ability of an individual to transmit one TSWV isolate was inherited as a recessive trait in the populations tested [22]. Viral proteins underlying thrips-TSWV interactions critical for transmission to occur have also been identified, but not related specifically to transmission by *T. tabaci* [6], [31]. The results of these studies suggest that genetic variation of thrips populations, virus isolates, or both thrips and isolates are important factors underlying the localized importance of this species as a vector of TSWV. However, no transmission study has been conducted with multiple TSWV isolates and multiple thrips populations to quantify the relative importance of vector and viral determinants underlying variation in transmission of TSWV.

This study was motivated by our specific need to understand the potential of *T. tabaci* as a vector in NC, and by the larger need to understand thrips-TSWV interactions that underlie variation in the importance of *T. tabaci* and potentially other species as vectors worldwide. Therefore, the objectives of this study were two-fold: 1) to quantify variation in transmissibility of TSWV isolates by *T. tabaci*, and the efficiency of *T. tabaci* to transmit isolates of TSWV in NC, and 2) to examine the possibility that genetic interactions and local adaptation contribute to the localized nature of this species as a vector of TSWV. *T. tabaci* collected from multiple locations in NC were used to establish isofemale lines that were tested for their ability to transmit multiple TSWV isolates collected at the same and different locations as the thrips. The data generated from these experiments show that the interaction of individual vector and virus isolates was an important factor underlying variation in transmission efficiency, and that on average, higher transmission rates occurred among sympatric vector-isolate pairings compared to allopatric vector-isolate pairings.

## Materials and Methods

### Ethics Statement

No specific permits were required for the described field studies. No permission was required to collect insects or plant material from Weeksville, Clayton, Kinston, Jackson Springs, or Mills River collection sites. These reside on university research farms (with whom we are affiliated) or unregulated public lands. Verbal permission was obtained from private land owners for the Cove City, Candor, Apex, and Faison collections sites, respectively. The field studies did not involve regulated, endangered, or protected species.

### Thrips Populations

**Figure 1:**
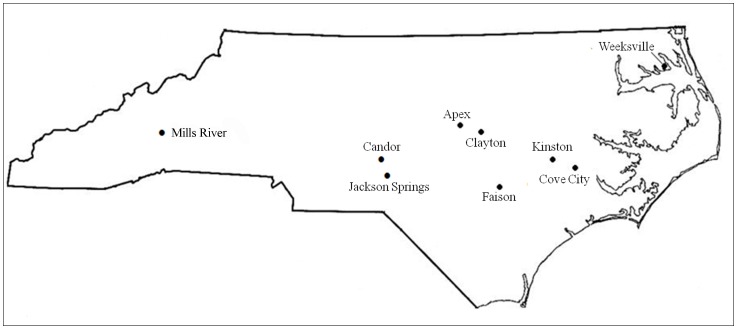
Map of collection locations for *Thrips tabaci* individuals and *Tomato spotted wilt virus* isolates.

*Thrips tabaci* individuals were collected from cultivated and weed plant hosts at 9 different locations in North Carolina in 2010 (Figure 1). Field collected thrips were kept separate, and individual females were used to establish isofemale lines; all individuals in an isofemale line originated from one female (Table 1). The reproductive mode of the isofemale lines was determined by sexing the offspring of unmated F1 offspring [32]. A total of 21 isofemale lines were established from females collected in 2010. Voucher specimens of isolines were deposited in the North Carolina State University Insect Museum. All but three of the field-collected female thrips used to initiate isofemale lines were thelytokous; virgin GJ3, MHC-1, and MHC-2 females, produced all male F1 offspring. However, these lines stopped producing males and began producing all female offspring parthenogenetically before experiments were initiated. Sex ratios have been shown to fluctuate seasonally and yearly in *T. tabaci* populations [32]–[34]. The cessation of male production in some laboratory colonies and inbred lines established from male producing individuals has also been observed [35] [Jacobson personal observation], as well as the opposite trend in which a seemingly thelytokous population begins to produce males [Nault, personal observation]. Due to the variations in male production previously described and the care taken to rear isofemale lines in isolation, the cessation of male production by these arrhenotokous lines was not believed to be due to contamination between isofemale lines. In addition, an isofemale line from a *T. tabaci* laboratory colony collected in 2009 and capable of transmitting TSWV in preliminary experiments was initiated and included in these experiments. After three generations in the laboratory, offspring from each isofemale line were used in the transmission experiments.

**Table 1:** Collection information for *Thrips tabaci* isofemale lines used in the transmission experiments.

Isofemale Line	Location	Host	Year	Reproductive Mode
Apex	Apex, NC: 35.718306N, −78.926981W	*Allium cepa*	2010	Thelytokous
SR3-1	Cove City, NC: 35.2533N, −77.2843W	*Raphanus raphanistrum*	2010	Thelytokous
IPOC	Cove City, NC: 35.2249N, −77.2757W	*Allium spp.*	2010	Thelytokous
KIN-09	Kinston, NC: 35.300N, −77.5763W	*Allium spp.*	2009	Thelytokous
KIN-1	Kinston, NC: 35.300N, −77.5763W	*Allium cepa.*	2010	Thelytokous
SH2	Jackson Springs, NC: 35.1938N, −79.6848W	*Secale cereale*	2010	Thelytokous
SH63	Jackson Springs, NC: 35.1921N, −79.6868W	*Allium spp.*	2010	Thelytokous
SH68	Jackson Springs, NC: 35.1799N, −79.6749W	*Allium spp.*	2010	Thelytokous
SH72	Jackson Springs, NC: 35.193N, −79.6819W	*Raphanus sativus* var. *niger*	2010	Thelytokous
COT1-2	Faison, NC: 35.1212N, −78.1582W	*Raphanus raphanistrum*	2010	Thelytokous
COT1-8	Faison, NC: 35.1212N, −78.1582W	*Brassica oleracea*	2010	Thelytokous
COT2-1	Faison, NC: 35.1223N, −78.131W	*Raphanus raphanistrum*	2010	Thelytokous
COT2-2	Faison, NC: 35.1223N, −78.131W	*Allium spp.*	2010	Thelytokous
COT2-4	Faison, NC: 35.1223N, −78.131W	*Allium cepa*	2010	Thelytokous
GJ3	Candor, NC: 35.2383N, −79.7693W	*Allium cepa*	2010	Arrhenotokous
GJ4	Candor, NC: 35.2383N, −79.7693W	*Allium cepa*	2010	Thelytokous
GJ6	Candor, NC: 35.2383N, −79.7693W	*Brassica oleracea*	2010	Thelytokous
GJ7	Candor, NC: 35.2383N, −79.7693W	*Brassica oleracea*	2010	Thelytokous
WEEK	Weeksville, NC: 36.1725N, −76.1295W	*Brassica oleracea*	2010	Thelytokous
MHC1	Mills River, NC: 35.4292N, −82.5614W	*Allium cepa*	2010	Arrhenotokous
MHC2	Mills River, NC: 35.4292N, −82.5614W	*Allium cepa*	2010	Arrhenotokous

### TSWV Isolates and Source Plants for Transmission Experiments

An isolate collected in 2009 that was capable of being transmitted by *T. tabaci* in preliminary experiments was included in these experiments. This isolate was maintained in the greenhouse on *Emilia sonchifolia* using alternating rounds of mechanical inoculation and transmission with *T. tabaci.* In addition, multiple TSWV isolates were collected from seven of the same locations as the thrips in 2010 (Figure 1, Table 2). A single field-collected leaf of an infected plant was used as source material to mechanically inoculate TSWV into *Emilia sonchifolia* seedlings at the second true leaf stage [36]. Inoculation buffer (10 mM Tris-HCl, pH 7.8, 10 mM Na_2_SO_3_, 0.1% cysteine-HCl) was used in all mechanical inoculations. Infected plants were then allowed to grow for 4–6 weeks in the greenhouse at which time they were used as source material to inoculate the *E. sonchifolia* seedlings that were used in the transmission experiments. All infected seedlings from the second mechanical inoculation were transplanted into individual pots, placed into thrips-proof screen cages made of 100 micron screen (Midwest Filter Corp., Lake Forest, IL) and allowed to grow for 2–3 weeks in the greenhouse before they were used in experiments. A total of 14 isolates were used in these experiments.

**Table 2:** *Tomato spotted wilt virus* isolate sample collection information.

Isolate	Location	Host	Year
Ap-1	Apex, NC: 35.718306N, 78.926981W	*Solanum lycopersicum*	2010
Ap-2	Apex, NC: 35.718306N, 78.926981W	*Solanum lycopersicum*	2010
Ap-3	Apex, NC: 35.718306N, 78.926981W	*Solanum lycopersicum*	2010
Ap-4	Apex, NC: 35.718306N, 78.926981W	*Solanum lycopersicum*	2010
SR3-1	Cove City, NC: 35.2523N, −77.2849W	*Nicotiana tabacum*	2010
SR3-2	Cove City, NC: 35.2523N, −77.2849W	*Nicotiana tabacum*	2010
SR3-3	Cove City, NC: 35.2523N, −77.2849W	*Nicotiana tabacum*	2010
Am-1	Cove City, NC: 35.2514N, −77.5811W	*Nicotiana tabacum*	2010
KIN-09	Kinston, NC: 35.300N, −77.5763W	*Nicotiana tabacum*	2009
KIN-1	Kinston, NC: 35.300N, −77.5763W	*Nicotiana tabacum*	2010
SH-1	Jackson Springs, NC: 35.1799N, −79.6749W	*Nicotiana tabacum*	2010
SH-2	Jackson Springs, NC: 35.1799N, −79.6749W	*Nicotiana tabacum*	2010
SH-3	Jackson Springs, NC: 35.1799N, −79.6749W	*Nicotiana tabacum*	2010
SH-P	Jackson Springs, NC: 35.1921N, −79.6868W	*Capsicum annuum*	2010
COT-P	Faison, NC: 35.1223N, −78.131W	*Capsicum annuum*	2010
GJ	Candor, NC: 35.2383N, −79.7693W	*Solanum lycopersicum*	2010

Disease-free *E. sonchifolia* leaf discs were used as indicator tissue in the transmission experiments. All of these plants were grown in thrips-proof greenhouses under natural daylight until they were used in the experiments. Leaf discs were cut from leaves of mature plants with a #7 cork borer and placed into 50 mm x 9 mm petri dishes with tight fit lids (Becton, Dickinson and Company, Mississauga, Ontario, Canada) that were lined with moist filter paper. Leaf discs were cut the same day they were infested with thrips.

### Transmission of TSWV by Thrips tabaci

A total of 89 isolate x isofemale line combinations were tested. Due to the size of these experiments, this protocol was repeated five times; 2–3 isolates were used with multiple isofemale lines during each time period. Each isofemale line was characterized for its ability to transmit 1–5 of the other isolates collected, depending upon availability of thrips and TSWV infected plants. All but three isofemale lines were tested for their ability to transmit the isolate collected from the same location as the thrips that was used to establish the isofemale line. In the case of these three exceptions, no sympatric virus isolate was collected.

TSWV infected *E. sonchifolia* plants individually housed in a thrips-proof sleeve cage were each infested with 5 adult females from a single isofemale line. A total of five TSWV infected plants were infested with thrips from each isofemale line. These plants were kept in a climate controlled room at 25°C with 50% RH and a 14∶10 L:D cycle. Females were allowed to feed and reproduce on the plant for 11–12 days, after which the leaves of the plants were removed and placed into a small cup with uninfected cabbage. 1–2 days after the offspring eclosed to adults they were confined individually with disease-free *E. sonchifolia* leaf discs (described above) and held at 25°C for 48 hours. At least 10 individuals from an isofemale line were used to test for transmission of TSWV. If adults were not collected the 1^st^ day, another attempt to collect adults occurred 3 days later. No adults were collected after that to avoid collecting offspring that did not have a sufficient acquisition access period. Each adult collected was confined with a single *E. sonchifolia* leaf disc for 48 hours after which the adult was removed. The leaf discs were held another 3 days before being tested for TSWV with DAS-ELISA (Agdia, Inc., Elkhart, IN, USA). Four negative controls (uninfected *Emilia sonchifolia*), 2 negative buffer controls and one positive control were included in each ELISA plate. Samples with a reading higher than the mean +4 standard deviations from the control samples were considered to be TSWV positive.

### Data Analysis

Binary transmission data were analyzed using SAS Software, Proc Glimmix (SAS Institute, Cary, NC). Mean transmission rates were calculated for 89 isolate-isofemale line combinations, each isofemale line, and each isolate. The effects of virus isolate and isofemale line were examined for 88 isolate-isofemale line combinations, and were also examined by location where multiple isofemale lines and/or isolates were collected. Transmission data for MHC-2 isofemale line was excluded from these analyses because this line was not tested for its ability to transmit more than one isolate. The probability of TSWV transmission based on virus isolate, isofemale line, and their interaction was then analyzed first. An additional model was then run with virus isolate, isofemale line and sympatry variables as main effects to test for significant differences between sympatric TSWV isolate-isofemale lines (collected from the same location) versus allopatric TSWV isolate-isofemale lines (collected from different locations).

## Results

**Figure 2:**
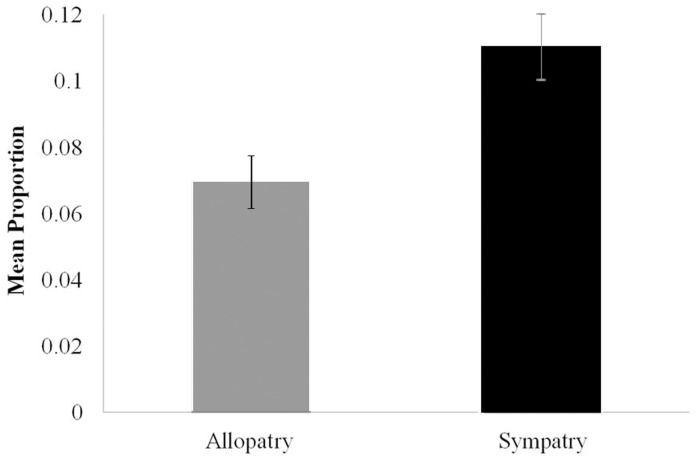
Mean (standard error) proportion of *Thrips tabaci* that transmitted *Tomato spotted wilt virus* to indicator tissue in sympatric vector-isolate pairings versus allopatric vector-isolate pairings were significantly different *(P* = 0.0143).

A large amount of variation in TSWV transmission was observed among the 20 isofemale lines tested for their ability to transmit 2–10 isolates (Table 3). The overall mean proportion of thrips transmitting TSWV for all transmission assays was 0.10, but ranged from 0–0.55 for different isolate-isofemale line combinations. No transmission occurred for some isolate-isofemale line combinations, however, every isofemale line transmitted at least one of the isolates, and each isolate was transmitted by multiple isofemale lines. There was extensive variation among virus isolates in their ability to be transmitted by individual isofemale lines and among isofemale lines in their ability to transmit different virus isolates. The proportion of thrips within a single isofemale line that transmitted TSWV varied up to 18 fold among virus isolates. Similarly, the proportion of *T. tabaci* that transmitted a single isolate varied up to 45 fold among different isofemale lines. The statistical analysis examining the effects of isolate, isofemale line, and their interaction on the probability of TSWV transmission showed that the main effect of isofemale line was not statistically significant (*P* = 0.0964), but the main effect of isolate (*P*<0.0001) and the interaction of isolate and isofemale line (*P* = 0.011) were both significant. To further investigate the significant interaction between thrips isofemale lines and virus isolates, another analysis was conducted that replaced the interaction term with the variable sympatry that defined vector-virus pairings as sympatric or allopatric. The results of this analysis showed that virus isolate (*P*<0.0001), isofemale line (*P*<0.0001) and sympatry (*P* = 0.0012) were all highly significant, and that, on average, sympatric vector-virus pairing resulted in higher transmission than allopatric pairings (Figure 2). These results indicate that the interaction of *T. tabaci* and isolate genetic determinants underlie successful transmission of TSWV by *T. tabaci*. In addition, higher transmission rates in sympatric vector-virus pairings suggest that local adaptation is occurring between *T. tabaci* and TSWV isolates.

**Table 3:** Mean proportion of *Thrips tabaci* isofemale line progeny transmitting *Tomato spotted wilt virus* (TSWV) to indicator leaf discs for each *Thrips tabaci* isofemale line-*Tomato spotted wilt virus* isolate combination tested, and overall mean proportion for each isolate and isofemale line.

	Isolate														
Thrips‡ Line	Ap-1	Ap-2	Ap-3	Ap-4	SR3-1	SR3-3	Am-1	Kin09	Kin	SH-2	SH-3	SH-P	GJ	Cot-P	Mean
Apex	–	**0.05** † **(0.05) N = 20**	–	–	–	0.10 (0.04) N = 58	0.06 (0.03) N = 66	0(0) N = 56	0(0) N = 65	0.06 (0.03) N = 79	0.05 (0.03) N = 110	–	–	–	0.04 (0.01)N = 454
SR3-1	0.03 (0.02) N = 79	–	–	0.06 (0.03) N = 50	–	**0.08 (0.03) N = 61**	0.03 (0.03) N = 36	0(0) N = 17	0(0) N = 36	–	0.11 (0.06) N = 27	–	–	–	0.05 (0.01)N = 306
IPOC	–	0(0) N = 11	0.06 (0.03) N = 53	–	–	0.07 (0.03) N = 54	**0.20 (0.05) N = 65**	0(0) N = 14	0.08 (0.03) N = 67	0.13 (0.06) N = 13	0.03 (0.02) N = 67	–	–	–	0.09(0.01) N = 344
KIN09	–	0.06 (0.03) N = 250	–	–	–	0.15 (0.05) N = 52	0.06 (0.03) N = 50	**0.08 (0.03) N = 72**	**0.13 (0.04) N = 55**	–	0.10 (0.04) N = 52	–	–	–	0.10 (0.02)N = 531
KIN1	–	–	0.02 (0.02) N = 49	–	–	0.08 (0.04) N = 42	0.21 (0.05) N = 66	**0.11 (0.05) N = 35**	**0.08 (0.03) N = 84**	–	0.01 (0.01) N = 68	–	–	–	0.09 (0.02) N = 344
SH2	–	0.20	0.06	–	0.03	0.06	0.21	0.03	0.15	**0.17**	**0.16**	**0.55**	–	–	0.13
		(0.12)N = 10	(0.03)N = 54		(0.02)N = 78	(0.02)N = 141	(0.07)N = 28	(0.02)N = 70	(0.05)N = 54	**(0.03)** **N = 120**	**(0.03)** **N = 131**	**(0.07)** **N = 52**			(0.01)N = 738
SH63	0.08 (0.03) N = 85	–	0.10 (0.04) N = 27	0.21 (0.05) N = 60	0.08 (0.03) N = 78	0.10 (0.03) N = 102	0.25 (0.06) N = 51	–	0.06 (0.03) N = 54	**–**	**0.05 (0.04) N = 21**	**–**	–	–	0.12 (0.01)N = 478
SH68	–	–	0.04 (0.02) N = 62	–	–	0.06 (0.05) N = 17	–	0(0) N = 13	0.17 (0.05) N = 48	**0.07 (0.05) N = 40**	**0.02 (0.02) N = 42**	**0.28 (0.07) N = 39**	–	–	0.10 (0.02)N = 261
SH72	–	–	–	–	–	0.20 (0.09) N = 20	0.10 (0.04) N = 63	–	–	–	–	–	–	–	0.12 (0.04)N = 83
COT1-2	–	–	–	–	–	–	–	–	–	–	–	–	0.45 (0.07) N = 51	**0.12 (0.04) N = 52**	0.28 (0.05)N = 103
COT1-8	–	–	–	–	–	–	–	–	–	–	–		0.12 (0.04) N = 48	**0.17 (0.05) N = 53**	0.14 (0.03)N = 101
COT2-1	–	–	–	–	–	–	–	–	–	–	–	–	0.03 (0.02) N = 51	**0.06 (0.03) N = 54**	0.04 (0.02)N = 105
COT2-2	–	–	–	–	–	–	–	–	–	–	–	–	0	**0.02**	0.01
													(0) N = 38	**(0.02) N = 56**	(0.01)N = 94
COT2-4	–	–	–	–	–	–	–	–	–	–	–	–	0.19 (0.05) N = 59	**0.06 (0.03) N = 51**	0.12 (0.03) N = 110
GJ3*	–	–	–	–	–	–	–	–	–	–	–	–	**0.15 (0.05) N = 34**	0.02 (0.02) N = 53	0.09 (0.03)N = 87
GJ4	–	–	–	–	–	–	–	–	–	–	–	–	**0.18 (0.06) N = 34**	0(0) N = 25	0.10 (0.04)N = 59
GJ6	–	–	–	–	–	–	–	–	–	–	–	–	**0** **(0) N = 52**	0.04 (0.03) N = 47	0.02 (0.01)N = 99
GJ7	–	–	–	–	–	–	–	–	–	–	–	–	**0.13 (0.05) N = 52**	0.02 (0.02) N = 47	0.08 (0.03)N = 99
WEEK	–	0.13 (0.05) N = 47	0(0) N = 52	–	–	0.12 (0.04) N = 75	0.06 (0.05) N = 17	0.04 (0.02) N = 69	0.04 (0.03) N = 49	–	0.14 (0.05) N = 42	–	–	–	0.08 (0.01)N = 351
MHC1	–	–	–	–	–	–	–	–	–	–	–	–	0.13	0	0.08
													(0.07) N = 23	(0) N = 17	(0.04)N = 40
*MHC2	–	–	–	–	–	–	–	–	–	–	–	–	0.28 (0.05) N = 43	–	0.28 (0.05)N = 43
Mean	0.05(0.02)N = 164	0.09 (0.02)N = 338	0.05 (0.01)N = 297	0.15 (0.03)N = 110	0.05 (0.02)N = 156	0.09 (0.01)N = 622	0.14 (0.02)N = 442	0.04 (0.01)N = 346	0.08 (0.01)N = 512	0.12 (0.02)N = 252	0.08 (0.01)N = 560	0.44 (0.05)N = 91	0.12 (0.01)N = 485	0.06 (0.01)N = 455	0.10 (0.01)N = 4830

‡ Isofemale line started from single field collected Thrips tabaci.

* Arrhenotokous isofemale lines.

† Bold values indicate sympatric isolate-isofemale line pairings.

Standard error is given in parenthesis and the number of individuals tested for each isofemale line-isolate combination, N, is given.

## Discussion

The results from this study corroborate previous reports of large amounts of variation in vector competence of *T. tabaci* occurring among different populations [16]–[25]. However, this is the first study to characterize this variation using more than two TSWV isolates, and with isofemale lines instead of colonies that vary in genetic composition. Our results show that the vector competence of *T. tabaci* varied among isofemale lines in an isolate specific manner. Similarly, the transmissibility of the isolates by *T. tabaci* varied among isolates in a manner that was isofemale line specific. The interaction between virus isolates and isofemale lines was an important factor in TSWV transmission. On average, higher transmission rates were seen among sympatric TSWV isolate-isofemale lines versus allopatric TSWV isolate-isofemale lines, which is suggestive of local adaptation between virus and vector. Our failure to observe this effect for all sympatric virus-vector pairings is not unexpected given the abundance and widespread occurrence of two other TSWV vectors, *F. fusca* and *F. occidentalis,* and their importance in the epidemiology of TSWV in NC cropping systems [12]–[14], [26], [37]. While local adaptation is observed for many study systems involving host-parasite interactions, detecting evidence for local adaptation between TSWV and *T. tabaci* among tobacco agroecosystems where *T. tabaci* populations are generally scarce but can be locally abundant and two other vectors (*F. fusca* and *F. occidentalis*) are generally present was somewhat surprising. Detecting local adaptation under these conditions supports the idea that there is intense selection pressure put on viruses by their vectors [38], and suggests that *T. tabaci* can influence local vector-virus dynamics despite the observed variation in transmission of TSWV and its generally low abundance.

Previous studies concluded that differences in transmission efficiency among populations of *T. tabaci* were due the presence of cryptic species groups that differ in their ability to transmit TSWV. It was believed that the group that reproduced with arrhenotokous parthenogenesis was capable of transmitting TSWV, while the groups that reproduced with thelytokous parthenogenesis were either not capable or very inefficient at transmitting TSWV [19], [28], [39]. In this study individuals with arrhenotokous and thelytokous reproduction were collected, however, there were not enough arrhenotokous populations collected to quantify potential differences that may exist between these groups. The thelytokous populations in NC transmitted with efficiencies that ranged from 0–45% indicating that the genetics for vector competence exist in these populations. This, coupled with the importance of specific thrips-isolate interactions underlying transmission, indicates that identification of the species group to which a *T. tabaci* population belongs to, based on characteristics such as reproductive mode, is not sufficient to make conclusions about their competency as a vector of TSWV.

Our results clearly demonstrate that the competency of *T. tabaci* as a TSWV vector is dependent upon the thrips and viral populations present in any given area. A better understanding of population structure and clonal distribution of *T. tabaci* may provide valuable insights on the abundance and distributions of populations that vary in their ability to transmit TSWV. In addition, the specificity of *T. tabaci*-TSWV isolate interactions underlying transmission provide a good model system for characterizing thrips vector components associated with vector competence. Using this system to investigate mechanisms responsible for vector competence of this species will help to improve our understanding of vector competence of thrips vectors of *Tospovirus* species, especially as new tospoviruses continue to emerge, and additional thrips species are identified as vectors [40]. Future studies in these areas will aid in the development of TSWV management programs where *T. tabaci* is present and provide fundamental information relevant to other insect-vectored plant and Bunyaviridae viruses.
